# Complete genome sequence of *Acinetobacter pittii*-infecting bacteriophage GUN

**DOI:** 10.1128/mra.00844-26

**Published:** 2026-09-18

**Authors:** Rita Domingues, Hugo Oliveira, Joana Azeredo, Martin J. Loessner, Enea Maffei

**Affiliations:** 1 CEB-Centre of Biological Engineering, University of Minho 56059, Braga, Portugal; 2 LABBELS–Associate Laboratory, Braga, Portugal; 3 Institute of Food, Nutrition, and Health, D-HEST, ETH Zurich 27219, Zurich, Switzerland; DOE Joint Genome Institute, Berkeley, California, USA

**Keywords:** *Acinetobacter pittii*, *Friunavirus*, capsule depolymerase, co-host enrichment, bacteriophage

## Abstract

Bacteriophage GUN infects *Acinetobacter pittii* and belongs to the genus *Friunavirus*. Genome analysis identified a 41,754 bp genome encoding 62 putative proteins, including a depolymerase. GUN was isolated using two *A. pittii* strains expressing distinct capsule types in co-culture.

## ANNOUNCEMENT

The *Acinetobacter calcoaceticus-baumannii complex* (ACB complex) is a group of major opportunistic nosocomial pathogens (1, 2). A thick capsular polysaccharide layer is commonly present in ACB complex strains, and strong selection pressures have generated more than 200 known K-types (3–5). Most bacteriophages infecting the ACB complex display a narrow host range because they rely on K-type-specific, tail-associated depolymerases that recognize and locally degrade the capsule, thereby enabling phage attachment and infection (6–11).

**Fig 1:**
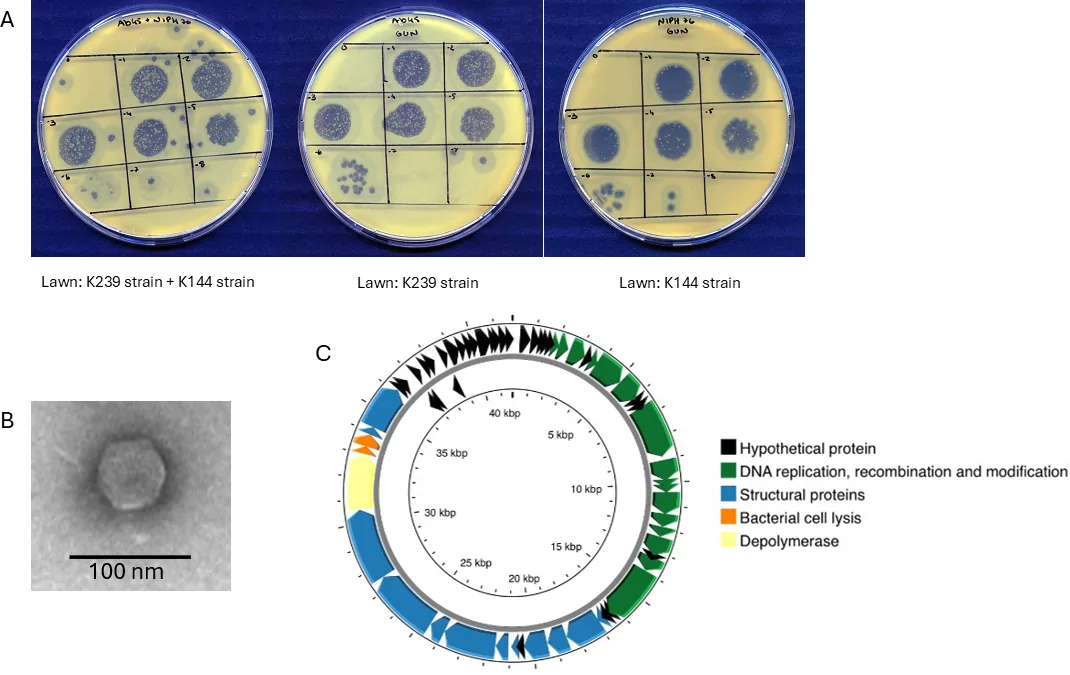
(**A**) Images of GUN phage plaques in lawns with both hosts (K239-strain and K144-strain) and with each host. (**B**) TEM image of GUN (scale bar, 100 nm) negatively stained with 2% uranyl acetate. (**C**) Genomic map of GUN (62 predicted proteins coloured according to their function) using the CGView.

Phage GUN was isolated from a sewage sample taken in Bern, Switzerland, in September 2024, using two *Acinetobacter pittii* strains as co-hosts: Ap45 (K239), previously isolated from a wound at Lausanne University Hospital (CHUV), and NIPH 76 (K144), obtained from Alexandr Nemec’s NIPH collection (5). Isolation followed previously described protocols based on phage precipitation and subsequent phage discovery in co-culture (Phage DisCo) (12–14). Briefly, viral particles present in an aqueous sample were precipitated using ZnCl_2_ at a final concentration of 40 mM and incubated at 37°C for 1 h. The suspension was centrifuged (8,000 × *g* for 10 min), and the resulting pellet was resuspended in 1 mL of SM Buffer. The suspension was mixed with 500 µL of Ap45 and NIPH76 overnight cultures together with tryptic soy broth (TSB) soft agar (0.6%), on a TSB agar plate, and incubated at 37°C for 16 h. Clear phage plaques were picked with sterile tips and purified three times onto freshly inoculated soft agar overlays of both host strains together. The purified phage formed plaques on Ap45 and NIPH 76, both individually and when the strains were combined in co-culture (Fig. 1A). Phage morphology analysis by transmission electron microscopy using a Jeol JEM 1400 with uranyl acetate staining, as previously described, revealed that GUN has a podovirus-like morphology (Fig. 1B) (15). Phage particles feature an isometric head of approximately 54 nm and a short non-contractile tail of approximately 7 nm in length (Fig. 1B). Genomic DNA was extracted from lysates (10^10^ PFU/mL) using the Norgen Phage DNA Isolation Kit following the manufacturer’s instructions. DNA was prepared for sequencing using the Illumina DNA Prep tagmentation Kit and was sequenced on an Illumina NextSeq 2000 platform using a 300-cycle flow cell kit to produce 2 × 150 bp paired reads, yielding a total of 1,722,478 paired reads. Reads were trimmed using BBDuk v38.37 to remove sequencing adapters and *de novo* assembled in Geneious Prime v.2019.2.3 (Biomatters Ltd.; https://www.geneious.com). This resulted in a genome assembly of 41,754 bp with a mean coverage of 55× and with a GC content of 39.2% (Fig. 1C). The genome was annotated with Pharokka v.1.3.2 (16), and manually curated using BLASTp (17) and HHpred (18) against the Pfam-A_v36 database (19). The presence of a depolymerase gene was determined using PhageDPO (20). All tools were run with default parameters. GUN was predicted to encode 62 proteins (Fig. 1C). Genome analysis using the taxMyPhage software (v.3.3.6) assigns GUN to the *Friunavirus* genus within the *Beijerinckvirinae* subfamily. Whole-genome BLAST (v.2.12.0) (17) against the National Center for Biotechnology Information Core Nucleotide Collection database (last update 18 July 2026) identified *Acinetobacter phage vB_ApiP_P2* (NC_042007.1) as the closest relative, with 95.87% nucleotide identity across 95% of the genome.

## Data Availability

The genome sequence for phage GUN and the associated reads was deposited in the European Nucleotide Archive under Bioproject accession number PRJEB113824. The reads used to generate the assembly have been deposited under the accession number ERR17422407. The assembly for phage GUN was deposited under the accession GCA_985694385.1. The *A. pittii* strain Ap45 sequencing reads and assembled contigs are provided under the BioProject accession number PRJNA750264. The *A. pittii* strain NIPH76 sequencing reads and assembled contigs are provided under the BioProject accession number PRJNA842650.
